# Correction: Threshold Games and Cooperation on Multiplayer Graphs

**DOI:** 10.1371/journal.pone.0152340

**Published:** 2016-03-21

**Authors:** Kaare B. Mikkelsen, Lars A. Bach

The affiliation for the second author is incorrect. Lars A. Bach is affiliated with: **1** Interacting Minds Center, Aarhus University, DK-8000 Aarhus C, Denmark, **3** Interdisciplinary Center for Organizational Architecture(ICOA), Aarhus University, DK-8210 Aarhus V, Denmark

The Data Availability statement for this paper is incorrect. The correct statement is: All relevant data are within the paper and Supporting Information files.
